# Effects of Mini-Spidroin Repeat Region on the Mechanical Properties of Artificial Spider Silk Fibers

**DOI:** 10.1021/acsomega.4c06031

**Published:** 2024-10-07

**Authors:** Benjamin Schmuck, Gabriele Greco, Olga Shilkova, Anna Rising

**Affiliations:** † Department of Medicine Huddinge, Karolinska Institutet, Neo, 141 83 Huddinge, Sweden; ‡ Department of Animal Biosciences, Swedish University of Agricultural Sciences, 750 07 Uppsala, Sweden

## Abstract

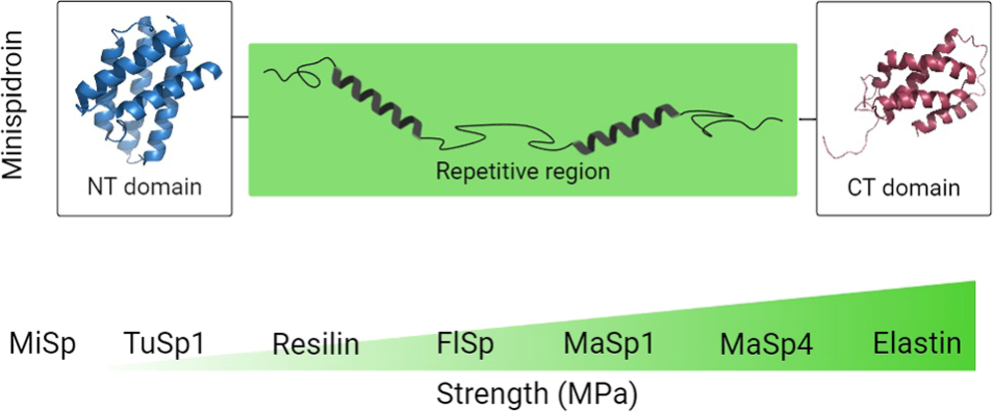

Spiders can produce up to seven different types of silk, each with unique mechanical properties that stem from variations in the repetitive regions of spider silk proteins (spidroins). Artificial spider silk can be made from mini-spidroins in an all-aqueous-based spinning process, but the strongest fibers seldom reach more than 25% of the strength of native silk fibers. With the aim to improve the mechanical properties of silk fibers made from mini-spidroins and to understand the relationship between the protein design and the mechanical properties of the fibers, we designed 16 new spidroins, ranging from 31.7 to 59.5 kDa, that feature the globular spidroin N- and C-terminal domains, but harbor different repetitive sequences. We found that more than 50% of these constructs could be spun by extruding them into low-pH aqueous buffer and that the best fibers were produced from proteins whose repeat regions were derived from major ampullate spidroin 4 (MaSp4) and elastin. The mechanical properties differed between fiber types but did not correlate with the expected properties based on the origin of the repeats, suggesting that additional factors beyond protein design impact the properties of the fibers.

## Introduction

Spider major ampullate silk is among the most impressive fibers known in terms of mechanical properties, which stem from a unique combination of high tensile strength and extensibility.^1−4^ In addition, spiders make up to seven distinct types of silk,^5,6^ each endowed with unique properties tailored to each type’s specific function.^7,8^ For instance, major ampullate silk possesses the highest strength and is used as a lifeline,^9^ aciniform silk is more extensible and used for prey wrapping,^10^ while flagelliform silk coated with aggregate glue is extremely extensible and hence used for making the capture spiral.^11^

All silk types are mainly composed of spider silk proteins (spidroins), which in general are very large with a molecular weight of up to 300 kDa.^12,13^ The spidroins share a common architecture, featuring a repetitive (Rep) region, which is flanked by globularly folded N-terminal (NT)^14^ and C-terminal (CT)^15^ domains. NT and CT are responsible for the high solubility of the spidroins during storage and for polymerization of the spidroins when exposed to shear and a decreased pH.^15−19^ The divergent mechanical properties of the silk fiber are likely to stem from disparities in the spidroins’ Rep region.^20−22^ For instance, the major ampullate spidroins (MaSps) carry characteristic poly-Ala blocks that alternate with Gly-rich regions,^23^ and according to the current model of the fiber structure–function relationship, the poly-Ala form nanosized β-sheet crystals in the mature fiber conferring the fiber strength,^24^ while the Gly-rich segments make up an amorphous and flexible matrix in which these crystals are embedded.^25,26^ Another example comes from the flagelliform silk which is composed of spidroins that have Pro-rich repeat regions, which are believed to contribute to the extreme extensibility of this silk type.^27^ Nevertheless, recent results suggest that this correlation is not straightforward, but that a combination of spidroins and other proteins is necessary to obtain the strength and extensibility of major ampullate silk.^28^

In the same way that spiders use their different silk types for specific purposes,^29^ fibers with different properties are needed for specific industrial applications. For instance, an important selection criterion of fibers for clothing is comfort,^30,31^ while sports goods could benefit from lightweight, elastic, and tough fibers.^32^ The diverse and unique properties of spider silks make the material attractive for many applications, for instance, biomedical^33−36^ and aerospace^37^ applications, as durable components in robotics,^38^ as sustainable wearables,^39^ in sports goods,^40^ and in other high-end textiles.^8,41^

Most artificial silk fibers are made from recombinant spidroin-like proteins that often consist of the Rep unit alone (no terminal domains). Fibers produced from large Rep proteins (300 kDa^42−44^) can feature GPa strength.^43,44^ However, this production method comes with the disadvantage that solvents such as hexafluoropropanol and methanol are needed during the spinning process and that the protein yield is rather low. Another strategy for producing artificial spider silk is to express natively folded mini-spidroins that consist of the NT, a short Rep, and a CT.^45−50^ The advantage of this approach is that the proteins can be purified using native conditions, and the artificial silk fibers are formed by decreasing the pH and/or using shear forces.^45−50^ This strategy, on the other hand, comes with the disadvantage that fibers are weaker, reaching a maximum strength of 250 MPa.^48,51^ One explanation for the reduced strength compared to the non-native spinning methodology could be the comparatively small size of the mini-spidroins of less than 100 kDa.^46−49,51,52^

**Figure 1:**
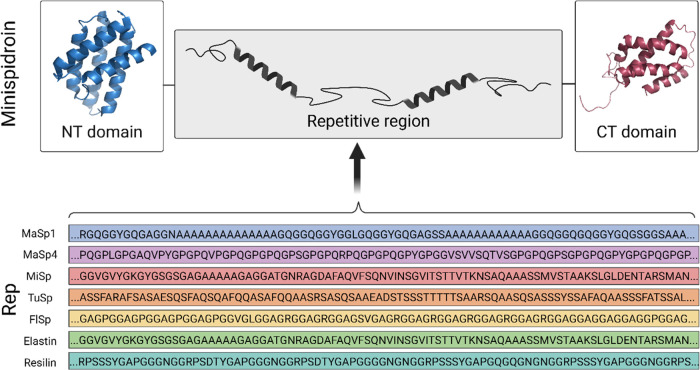
Using the basic mini-spidroin concept described by Andersson et al.,^45^ the Rep region in NT2RepCT was replaced with other natural and engineered repeats. Altogether 16 proteins were designed (listed in Table 1) with Rep regions originating from MaSp1 (three different constructs with an increased size of the Rep region and two with shortened poly-Ala blocks), MaSp4 (two constructs), elastin (two constructs), FlSp (two constructs), TuSp (two constructs), resilin (one construct), and MiSp (one construct). Examples of Rep regions are shown in the lower panel, the complete sequences of NT, CT, and all Rep regions are listed in Table S1, and a short description of each insert is provided in Table S2. The figure was made with Biorender.com using structures from Protein Data Bank (PDB)2MFZ and 4FBS.

With the aim to improve and specifically tailor the mechanical properties of artificial silk fibers made from mini-spidroins and to understand the relationship between the protein design and the mechanical properties of the fibers, we systematically modified the mini-spidroin NT2RepCT. NT2RepCT has a Rep from MaSp1 featuring two poly-Ala blocks located between NT and CT, expresses at high very yields of up to 21 g/L with *Escherichia coli*,^53^ and is extremely soluble in aqueous buffers (500 mg/mL).^54^ To modify NT2RepCT, we explored four different strategies: (1) to vary the poly-Ala segment length, (2) to increase the size of the MaSp1 region, (3) to insert Rep segments from different spidroin types, and (4) to insert Rep segments from other naturally occurring fibrous proteins. Thus, a battery of 16 mini-spidroins was designed (Figure 1), and fibers spun from these proteins were characterized by determining their mechanical properties.

## Results and Discussion

### Expression and Purification

In total, we designed 16 constructs that carry different Rep regions inserted between the terminal domains (complete sequences are listed in Tables S1 and S2). Three of these carried Rep regions from the MaSp1 from *Euprosthenops australis*, three from a MaSp4 from *Caerostris darwini*,^55^ two from elastin from *Homo sapiens*, two from a tubuliform spidroin (TuSp) from *Trichonephila clavipes*,^56^ two from a flagelliform spidroin (FlSp) from *T. clavipes*,^56^ one from resilin from *Drosophila simulans*,^57^ and one from minor ampullate spidroin (MiSp) from *Araneus ventricosus*.^58^ These proteins were named after the protein from which the repetitive segment was derived and the number of amino acid residues in the repeat region. Two additional constructs were based on the MaSp1 Rep region, in which the natural 15 and 14-residue-long poly-Ala stretches were replaced by four or eight consecutive Ala residues, which were named MaSp1_A_4_ and MaSp1_A_8_, respectively. As reference, already reported values for solubility and yield of the previously characterized NT2RepCT^51,53,54^ (according to the nomenclature described herein, NT2RepCT would be called MaSp1_77) and (A_3_I)_3_-A_14_^52^ proteins were used.

Most constructs expressed well and were soluble after cell lysis in a Tris-HCl buffer, reaching yields after purification of 26 and 250 mg/L in shake flask cultivations (Table 1 and Figure S1). Overall, lower yields after purification were obtained for mini-spidroins that carried longer Rep regions compared to shorter variants (Figure S2). This is in line with previous publications^46−48^ and could be due to the inability of the prokaryotic translational machinery to cope with the high demands of specific amino acid residues,^42^ increased aggregation propensity of long repetitive proteins, toxicity of the expressed proteins, and/or unfavorable secondary structure formation of the repetitive mRNAs.^59^

**Table 1:** List of Mini-Spidroins Studied in This Work^a^

strategy	construct	length of rep (# aa)	*M*_w_ (kDa)	solubility after cell lysis	yield after purification (mg/L)*	spinnability
control	NT2RepCT	77	33.2	+++	250^52^	++
	(A_3_I)_3_-A_14_	77	33.3	+++	207^52^	–
poly-Ala length	MaSp1_A_4_	56	31.7	+++	209	+
	MaSp1_A_8_	64	32.2	+++	170	–
MaSp1 length	MaSp1_110	110	35.7	+++	183	++
	MaSp1_173	173	40.7	n.a	0	n.a
	MaSp1_237	237	45.8	n.a	0	n.a
spidroin types	MaSp4_125	125	38.2	+++	231	++
	MaSp4_175	175	42.8	+++	80	++
	MaSp4_252	252	49.7	++	98	–
	MiSp_206	206	42.1	+++	114	++
	FlSp_132	132	36.8	++	157	++
	FlSp_232	232	44.7	+++	61	–
	TuSp1_174	174	43.2	+++	114	++
	TuSp2_345	345	59.5	+++	35	–
natural repeats	Resilin_142	142	39.5	++	48	++
	Elastin_116	116	36.5	+++	88	++
	Elastin_221	221	45.5	++	26	–

a A compilation of 18 mini-spidroins including two previously characterized proteins (NT2RepCT^51,53,54^ and (A_3_I)_3_-A_14_^52^). Expression values for NT2RepCT and (A_3_I)_3_-A_14_ were reported earlier by Arndt et al.^52^ *Average of a 10 × 1L cultures. Solubility after cell lysis was rated according to the following thresholds: + ++ almost all; + + more than 50%; + less than 50%; – not soluble; n.a. since no protein was expressed. The spinnability of the different constructs was rated as – not spinnable in that fibers were weak and fractured either when the fiber exited the capillary or while picking it up from the bath and guiding it to the collection wheel. + Spinnable, but discontinuous. To collect enough fibers for tensile testing, the fiber had to be picked up several times and guided to the collection wheel. + + Continuous spinning. The fiber was picked up and continuously collected for 1–2 min yielding up to 60 m of fiber, before the process was aborted to ensure evenly spaced fibers on the collection wheel.

### Spinning of Artificial Spider Silk

The mini-spidroins that could be isolated in a soluble form were concentrated and subjected to spinning using a protocol developed for NT2RepCT.^51^ This protocol is purely aqueous-based but differs slightly compared to previous studies published by our group^52,54^ in terms of buffer in the coagulation bath, reeling speed, and the tip diameter of the capillary used for extrusion of the spinning dope (see the Materials and Methods section for details). Using these spinning conditions, the different mini-spidroins were characterized as being continuously spinnable, discontinuous, or not spinnable. Spinnable fibers could be guided through the bath and collected onto a wheel rotating at 36 m/min. Some mini-spidroins formed fibers that were too fragile to be collected onto the rotating wheel and were then categorized as nonspinnable. Of the 18 mini-spidroin variants (16 novel + 2 controls), 10 were possible to spin into continuous fibers, seven were nonspinnable, and one type could be collected onto the wheel, but the spinning was not continuous (Table 1).

### Mini-Spidroins with Different Lengths of the Poly-Ala Blocks

The poly-Ala blocks found in the MaSp Rep region form crystals in the fiber, which are believed to confer the strength of the fibers. Molecular simulations have suggested that restricting the size of the crystals is imperative for optimizing the strength since large crystals will be more likely to bend and fracture than smaller ones.^24,60^ At the same time, poly-Ala stretches composed of less than seven residues are not expected to form β-sheet crystals.^61^ To build on these results, Hu and co-workers investigated recombinant spidroins containing 5, 8, or 12 Ala residues in the poly-Ala motif.^62^ They found that the construct containing 12 Ala residues produced artificial silk fibers that were weaker compared with the other constructs. The eight Ala spidroin produced the strongest (623 MPa) and toughest (107 MJ m^–3^) artificial silk fibers. Accordingly, we designed two variants of NT2RepCT in which the poly-Ala blocks were shortened to eight or four residues, respectively (MaSp1_A_8_ and MaSp1_A_4_). Surprisingly, MaSp1_A_8_ was not spinnable, as fibers broke when guided through the spinning bath. Likewise, it was difficult to spin and collect MaSp1_A_4_ fibers, which is reflected in the mechanical properties of these fibers which were among those with the lowest strength and extensibility of all fibers investigated herein (Table 2). Hence, reducing the number of Ala residues in the poly-Ala motif of NT2RepCT did not lead to improvement of the fibers’ mechanical properties.

**Table 2:** Mechanical Properties of Spinnable Constructs in Order according to the Strength^a^

construct	diameter (μm)	strain at break (%)	eng. strength (MPa)	Young’s modulus (GPa)	toughness modulus (MJ m^–3^)
MiSp_206	11.8 ± 0.6	93% ± 43%	74 ± 8	2.5 ± 0.4	59 ± 27
MaSp1_A_4_	17.7 ± 6.3	6% ± 2%	75 ± 28	2.0 ± 1.0	2 ± 1
TuSp1_174	19.8 ± 3.4	6% ± 2%	76 ± 19	2.3 ± 0.6	3 ± 1
Resilin_142	13.5 ± 3.9	21% ± 26%	97 ± 18	2.7 ± 0.8	17 ± 24
FlSp_132^b^	9.9 ± 3.0	93% ± 43%	98 ± 38	2.7 ± 0.8	74 ± 49
MaSp1_110	9.7 ± 1.7	106% ± 32%	105 ± 25	2.6 ± 0.9	75 ± 23
NT2RepCT^a^	8.8 ± 2.7	113% ± 28%	110 ± 30	2.2 ± 0.6	90 ± 30
MaSp4_125^b^	9.0 ± 2.9	89% ± 26%	134 ± 38	2.4 ± 0.6	84 ± 35
MaSp4_175	9.8 ± 2.5	83% ± 31%	143 ± 78	3.3 ± 1.6	96 ± 61
Elastin_116^b^	9.8 ± 2.9	105% ± 32%	143 ± 32	2.9 ± 1.1	109 ± 47

a For each construct, at least 10 fibers were tensile tested. Outliers were not removed. The values reported after ± represent one standard deviation. Representative stress–strain curves are shown in Figure S2. ^a^Added for comparison purposes where the values represent the average from 89 fibers tensile tested that originate from 8 different spinning occasions, as reported in Schmuck et al.^51^^b^The values are averages of fibers made from two or more spinning occasions and tensile testing of more than 20 fibers. A graphical representation and statistical significance compared to NT2RepCT is shown in Figure S4. Representative stress–strain curves for all fiber types, except for NT2RepCT, are shown in Figure S5. Representative stress–strain curves for NT2RepCT are shown in Schmuck et al.^51^

### MaSp1 Mini-Spidroins with Different Lengths of the Rep Region

To investigate the impact of longer repeat regions, we extended the repeat region of NT2RepCT so that the resulting mini-spidroins encompassed three poly-Ala blocks (MaSp1_110), five poly-Ala blocks (MaSp1_173), or seven poly-Ala blocks (MaSp1_237). We first assessed the expression levels and benchmarked against NT2RepCT that can be expressed and purified at high yields reaching 250 mg/L in shake flask cultivations.^52,54^ From shake flask cultures, MaSp1_110 can be obtained at 183 mg/L, but surprisingly, we could not identify the longer MaSp1_173 and MaSp1_237 on an SDS-PAGE of the cells after expression (Figure S3), which indicates a very low or nonexisting expression. Interestingly, others such as Heidebrecht and co-workers succeeded in expressing constructs having 24 poly-Ala blocks with a standard *E. coli* strain, showing that it is in principle possible to recombinantly express spidroins with longer Rep.^63^ In this particular example, each poly-Ala block hosted only 5 Ala residues, which is much fewer compared to the up to 15 consecutive Ala residues found in our MaSp1 Rep segment. Nevertheless, we believe that the longer poly-Ala block is probably not the only cause for the failed expression of MaSp1_173 and MaSp1_237, since a recent study by Hu et al. succeeded to express spidroins having 13 repeats where each repeat hosted a block of 12 Ala residues with an expression level of 2.5 g/L using a bioreactor.^62^ As far as we could judge, the authors in the two examples did not use any additional vectors during expression with *E. coli* to elevate the alanyl- or glycyl-tRNA pool.^42^ An additional factor that could explain the loss of expression would be if the longer mini-spidroin variants are toxic to the bacteria, but the exact reason for the abrupt drop in protein expression is not known. The expression system presented here could potentially be improved by screening different bacterial strains and promotors, using a different codon optimization strategy, and possibly also by designing different mini-spidroin variants.

Next, we attempted to spin fibers from MaSp1_110, which was successful. The mechanical characterization of these fibers showed no significant difference compared to that of NT2RepCT fibers (Table 2 and Figure S4). This may be a result of that the MaSp1_110 Rep region is not long enough to mediate a significant impact on fiber mechanical properties.

In theory, longer repeat regions should result in an increased number of intermolecular interactions and, thereby, stronger fibers. In line with this, several reports in the scientific literature investigated this aspect, applying all-aqueous and native conditions for expression, purification, and spinning without the use of denaturing agents in any step of the process. Zhou et al.^48^ found a strong dependency of strength and size when inserting aciniform spidroin repeat segments into a mini-spidroin. When such a small mini-spidroin was spun (45.8 kDa), the resulting fibers had a strength of 52 MPa, but when the repetitive segment was extended, which resulted in a 104 kDa construct, the resulting fibers had a strength of 245 MPa. Even though these results are quite impressive, the expression was overall low, and the larger 104 kDa variant was not soluble in aqueous buffers. In a follow-up study, 44 and 96 kDa recombinant spidroins with repeats from FlSp were spun into fibers. Interestingly, despite the relatively large difference in molecular weight, the fiber strength was moderately increased from 182 to 253 MPa using larger spidroins. At the same time, these fibers were rather brittle with a strain at break of 12%, which is surprising considering the impressive extensibility of the flagelliform silk.^46^ In the literature, there are also conflicting reports regarding the relationship between *M*_w_ and the mechanical properties of the fibers. In a study where spidroins containing different lengths of the MaSp1 repeat region were spun into fibers, the largest constructs (60.8 kDa) gave inferior fibers compared to a 42.5 kDa variant, which could be spun into a fiber with a strength of 149 MPa.^47^ The emerging inconclusive picture of the effect of longer Rep segments on the mechanical properties could be a result of numerous parameters, which are discussed further below.

Since we were unsuccessful in expressing the mini-spidroins based on MaSp1 from *Euprosthenops australis* with repeat regions exceeding 110 residues, we attempted to produce longer variants with repeats originating from other spidroins.

### Mini-Spidroins with Rep Regions from Different Spidroin Types

The mechanical properties of different spider silks vary significantly, and this is related to differences in the primary structure of the Rep region.^22,64^ As mentioned before, the major ampullate silk, made up primarily of MaSps,^65,70^ is the strongest of the different silks.^66^ The MaSp4 has been identified in the spider species *Caerostris darwini*(55,68) and *Araneus ventricous*(67) and has been associated with an increased extensibility of the fiber.^55^ To examine the influence of MaSp4 Rep on our artificial silk is especially interesting because this spidroin type has been scarcely studied. MiSps are the main constituents of the minor ampullate silk, which have a lower tensile strength than major ampullate silk, but increased extensibility. Flagelliform silk stands out as the most extensible fiber (>200%) of all of the different silk types which relates to the Pro-rich repeat region of the FlSps, while tubuliform silk is intermediate in terms of both strength and strain.^66^ Thus, we expected that the insertion of different Rep between NT and CT would make the artificial silk fibers substantially different compared to reference NT2RepCT fibers, even more so as we have previously seen a drastic effect by introducing only very few mutations in the Rep of NT2RepCT.^52^

The new mini-spidroins containing Rep from different spidroin types could all be successfully spun into fibers (Table 2), except for constructs having more than 220 residues in Rep (FlSp_232, MaSp4_252, and TuSp2_345). The longest constructs were classified as not spinnable because the fibers were too fragile to be collected or because of aggregate formation in the spinning dope, as in the case of TuSp2_345, for which extrusion was inhibited due to clogging. Among the eight mini-spidroin variants with Rep regions from different spidroin types, only MaSp4_125 and MaSp4_175 were significantly stronger. These fibers had an average strength of 134 and 143 MPa, respectively, compared to 110 MPa for NT2RepCT (Table 2 and Figure S4). Since NT2RepCT, MaSp4_125, and MaSp4_175 have similar *M*_w_ and identical terminal domains, the increase in strength could be related to the nature of the MaSp4 repeat. The results are surprising since the MaSp4 is Pro-rich and void of poly-Ala blocks, which should give a more extensible fiber according to the current structure–function models. Fibers obtained from the mini-spidroin constructs MiSp_206, and TuSp_174 were significantly weaker. The strain at break for all constructs with Rep from different spidroins was above 83%, which is comparable to NT2RepCT fibers. An exception is the construct TuSp1_174 which resulted in brittle fibers. In summary, we did not achieve mechanical properties that correlated to the behavior of the native silk fibers that the respective repeats were derived from, and moreover, the mechanical properties of the fibers made from the new mini-spidroins were very similar to the mechanical properties of NT2RepCT fibers.

### Mini-Spidroins with Rep Regions from Other Fibrous Proteins

In nature, the elastomers tropoelastin and resilin stand out as examples of proteins that are found in tissues with impressive mechanical properties. Tropoelastin is found in connective tissues and is cross-linked via Lys residues into elastin.^71^ Silk-elastin hybrid materials are frequently described in the literature in the context of making biomaterials, for instance, to assemble tissue scaffolds by electrospinning,^72^ to tune the mechanical properties of hydrogels,^73^ and for modulating the self-assembly kinetics of silk/elastin hybrids to make nanomaterials.^74^ Herein, we could efficiently produce and spin fibers from a 36.5 kDa silk-elastin hybrid protein (Elastin_116), but the longer variant of this protein (Elastin_221) could not be spun into continuous fibers, as was also the case for the longer constructs with Rep from other spidroins. Notably, the fibers spun from Elastin_116 proteins were the toughest among all fibers investigated herein, reaching 143 MPa in strength, a strain at break of 105%, a Young’s modulus of 2.9 GPa, and a toughness modulus of 109 MJ/m^3^.

Resilin is found in elastic tissues of insects and arthropods and the proteins are cross-linked via Tyr residues.^75,76^ Resilin and resilin/silk hybrids have been mainly investigated for their use as biomaterials,^77^ but there are also attempts to increase the mechanical properties of silkworm silk by inserting the resilin gene into the silkworm genome.^78^ In our hands, fibers made from a mini-spidroin carrying Rep from Resilin (Resilin_142) could be continuously spun. The strength of Resilin_142 fibers was within the experimental error compared to NT2RepCT fibers, but the strain at break was significantly reduced (Figure S4 and Table 2).

To our knowledge, there are no previous examples of silk-elastin or silk-resilin hybrid proteins that have been continuously wet-spun into macroscopic fibers. Silk-resilin/elastin hybrid proteins could in theory harness the high deformability and resilience of the respective natural materials, but to achieve this, we likely have to find ways to allow the repeat regions to fold properly and to achieve the intermolecular cross-links that are present in the native materials.

### Control Proteins

As already mentioned before, the spinning condition used in this study were optimized for NT2RepCT^51^ and have not been tested on the engineered variant (A_3_I)_3_-A_14_, which has been shown to form fibers that are as tough as native spider silk in a recent study.^52^ Interestingly, the (A_3_I)_3_-A_14_ was in this study categorized as nonspinnable and is therefore not listed in Table 2, because the fiber broke as soon as it was placed on the collection wheel. This is likely due to that the spinning conditions used herein were different from those in Arndt et al.^52^ Specifically, the glass capillaries used in the current study had a larger opening, the spinning bath did not contain NaCl, and the speed of collection was much faster. These results attest to previously published notions, that in order to achieve the best possible mechanical properties of a fiber spun from a specific mini-spidroin, the spinning conditions need to be screened and optimized systematically.^51^ However, performing such large screens for the relatively large number of proteins investigated herein would require several years’ work.

### General Discussion

The main intention of the present study was to investigate if and how different primary structures of the Rep region in mini-spidroins affect the mechanical properties of artificially spun spider silk when using all-aqueous spinning conditions. To our surprise, the strength of all as-spun fibers using identical spinning conditions was found to be within a rather narrow range of 74–143 MPa. Interestingly, the only constructs that were better in terms of strength compared to the control mini-spidroin NT2RepCT (MaSp1_77) are constructs based on MaSp4 (up 143 MPa)^55^ and elastin (143 MPa) (Table 2). None of the fibers developed herein had a higher strain at break than the control NT2RepCT (113%), but the majority of the constructs (73%) resulted in fibers with a strain at break above 50%. Only the MaSp1_A_4_ and TuSp1_174 fibers failed at the yielding point. The fiber with the highest strength that was significantly different compared to NT2RepCT was made from Elastin_116. The strain at break values of NT2RepCT and Elastin_116 fibers were comparable, as was the toughness modulus (Figure S4). We therefore conclude that the mechanical properties of fibers spun from mini-spidroins with diverse primary structures in their repeat regions are surprisingly similar. The relatively small improvements we could achieve in this cohort of proteins are in the range that can be achieved when changing the spinning conditions.^51^

**Figure 2:**
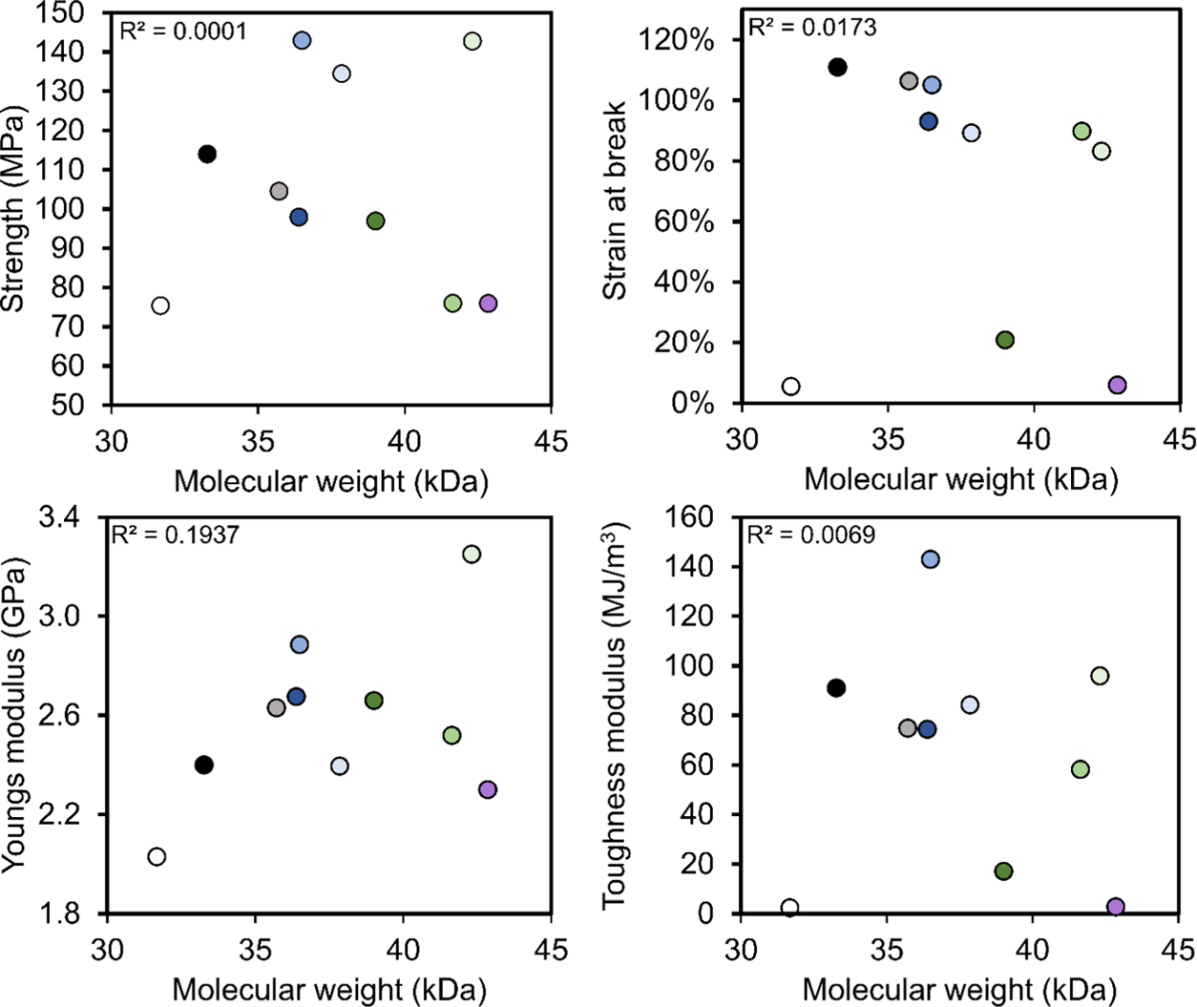
Mechanical properties for artificial silk fibers plotted against the molecular weight of the mini-spidroins used in this study. The different colors of the circles are used to represent the different mini-spidroins where white represents MaSp1_A_4_, black NT2RepCT, gray MaSp1_110, dark blue FlSp_132, blue Elastin_116, light blue MaSp4_125, dark green Resilin_142, green MiSp_206, light green MaSp4_175, and purple TuSp1_174. R^2^ was obtained by least-squares regression.

The question remains why most mini-spidroins investigated in this study did not give rise to fibers with more diverse mechanical properties and failed to reflect the properties of the respective native silk fibers. One explanation for this observation could be that the spinning conditions were not optimized for the respective construct, which could have substantially different optima concerning the buffer type, buffer concentration, pH, extrusion rate, and dope concentration. The biology of all glands, except the major ampullate gland, remains largely unexplored, which means that there may be unknown conditions or components that are important for the correct polymerization of a specific silk type. Specifically, several studies have shown that there are several nonclassical spider silk proteins in the major ampullate silk, all of which have unknown functions.^65,70,69^ These proteins and the combination of different proteins could potentially be important for the mechanical properties. However, even though the presence of other spidroins and nonspidroins should not be neglected,^65,70^ the bulk of each fiber type is made from the corresponding spidroin type,^70^ and thus they should be a main contributor to the mechanical properties. Another factor to take into account could be that the spidroin terminal domains may have evolved to function optimally with a specific repeat, which could also influence the results as we used the same NT and CT in all protein constructs. Yet another contributing factor could be that the repeat regions affect the ability of the mini-spidroins to form liquid–liquid phase-separated (LLPS) droplets before polymerization, which has been suggested to be an important step in the spinning process and deserves further investigation.^50,79−82^ Moreover, shear forces are important for alignment and to induce the formation of β-sheets,^83^ but the extrusion of the spidroins through the glass capillary used herein may not be enough to align the larger spidroins, thus causing a suboptimal arrangement and as a consequence a weaker fiber is obtained. The significantly smaller size of the mini-spidroins compared to the native spidroins could of course also affect the properties of the fiber if they are too short to form correct intermolecular interactions (Figure 2). When using denaturing conditions for the preparation of the spinning dope, the literature suggests that spidroins with an *M*_w_ comparable to native spidroins (up to 300 kDa) are required for making fibers with a strength of 1 GPa.^42,43,63,84^ Considering this, it may not be too surprising that we could not improve the strength more by spinning longer proteins, as the largest spinnable spidroin in this study had an *M*_w_ of only 43 kDa. Although our results suggest that there is no correlation between strength and molecular weight (Figure 2), it should be noted that molecular weight difference between the smallest and largest spidroin is only 37%, which may be too little to detect effects of protein molecular weight.

## Conclusions

We have shown that the basic NT-Rep-CT construct can host a broad range of different amino acid sequences in the Rep region and still maintain the ability to be spun into continuous fibers in our all-aqueous spinning system. Interestingly, we show that the primary structure of the Rep has a rather moderate influence on the resulting fibers’ mechanical properties, at least for repeat regions ranging from 32 to 43 kDa. We also found that spidroins with a molecular weight >45 kDa were difficult to spin in the spinning setup used in this work. Moving forward, optimizing protein design, spinning methodologies, and postprocessing techniques will be crucial for realizing the full potential of artificial spider silk in various applications.

## Materials and Methods

### Cloning and Protein Expression

Synthetic fragments were cloned in the pT7-His_6_-NT-CT vector between *Eco*RI and *Bam*HI sites. Resulted construct was transformed in BL21(DE3) *E. coli*, and the positive colonies were sequenced before preparing a glycerol stock, which was stored at −80 °C. For protein expression, LB broth medium containing kanamycin (70 μg/mL) was first inoculated with a glycerol stock of the previously transformed *E. coli* and then incubated overnight at 30 °C while shaking at 250 rpm. The overnight culture was used for inoculation of up to 10 × 1 L of fresh LB media also containing 70 μg/mL kanamycin. Then, the cultures were incubated at 30 °C with shaking (110 rpm) until OD_600_ reached 0.6, after which the temperature was lowered to 20 °C and protein expression was induced at OD_600_ 0.8 by adding isopropylthiogalactoside to a final concentration of 0.15 mM. The cells were cultured overnight at 20 °C with shaking (110 rpm) and were then harvested by centrifugation for 20 min at 5000*g* at 4 °C. The pellets were resuspended in 20 mM Tris pH 8 and stored at −20 °C.

### Protein Purification

Lysis was performed in a cell disrupter (T-S Series Machine, Constant Systems Limited) at 30 kPsi, after which the lysate was centrifuged at 25 000*g* at 4 °C for 30 min. The supernatant was loaded on 2 sequentially connected 20 mL of HisPrep FF16/10 (Cytiva) columns, washed with 5 CV 20 mM Tris-HCl pH 8.0 and 5 CV 2 mM imidazole in 20 mM Tris-HCl pH 8.0. The protein was eluted with 200 mM imidazole in 20 mM Tris-HCl pH 8.0. The eluted protein was dialyzed against 20 mM Tris (pH 8.0) at 4 °C overnight using a Spectra/Por dialysis membrane with a 6–8 kDa molecular-weight cutoff. SDS–PAGE (4–20%) and Coomassie Brilliant Blue staining were used to determine the purity of the protein. Broad Range Protein Ladder (Thermo Fisher Scientific) was used as a size standard. Protein concentration was determined by recording the absorbance of a 10-diluted dialyzed eluate at 280 nm.

### Preparation of the Spinning Dope

Purified protein was concentrated to ∼300 mg/mL with an Amicon Ultra-15 centrifugal filter unit (Merck-Millipore, Darmstadt, Germany) equipped with an ultracel-10 membrane (10 kDa cutoff) at 4000*g* and 4 °C to prepare the spinning dope. The concentration of the concentrated protein was determined by recording the absorbance at 280 nm of a 500 × diluted sample, in triplicate, before the spinning dope was transferred to a 1 mL syringe with a Luer lock (BD, Franklin Lakes, New Jersey), and stored at −20 °C.

### All-Aqueous Spinning of Artificial Silk Fibers

The all-aqueous spinning of the protein concentrate (spinning dope) was performed according to a method described by Andersson et al.^45^ and fine-tuned by Schmuck et al.^51^ Briefly, the spinning dope was thawed at room temperature, and the 1 mL syringe was connected to a pulled glass capillary with a tapered opening of 40–100 μm (G1 Narishige, Tokyo, Japan with an O.D. of 1.0 mm and μD of 0.6 mm, pulled using a Micro Electrode Puller, Stoelting Co. 51217, Wood Dale, Illinois). Then, the syringe was placed into a neMESYS low-pressure (290 N) syringe pump (Cetoni, Korbußen, Germany). Using a flow rate of 17 μL/min, the dope was extruded through the glass capillary into an 80 cm long coagulation bath containing 0.75 M acetate buffer (pH 5). If possible, the fiber was collected continuously at the end of the bath, using a rotating wheel, with a circumference of 35 cm, and a reeling speed of 59 cm s^–1^, at a relative humidity (RH) of <40%.

### Tensile Testing

Tensile testing was conducted with an Instron 5943 equipped with a 5N load cell. The fibers were mounted in a 1 cm × 1 cm paper frame using double-sided tape (circa 1 cm gauge length). The diameter of the fibers was determined with light microscopy by measuring the diameter three times, in three arbitrary locations along the fiber. Then, the paper frame was mounted in the tensile tester using grips before cutting the sides of the paper frame. During the tensile test, a displacement speed of 6 mm/min was applied. All tensile tests were conducted at an RH below 40% and at a temperature of 22 °C. The average diameter was used to calculate the cross-sectional area assuming a round cross section, which likely underestimated the strength of the fibers by a factor of 1.8.^86^ With the cross section and the gauge length, we calculated engineering stress and strain. The toughness modulus was calculated as the area under the stress strain curve and the Young's modulus as the slope in the initial linear elastic region of the stress strain curve.

## Supporting Information Available

SDS-PAGE images highlighting the expression and purification process of the spidroins; graphical representation of the mechanical properties of the fibers; stress–strain curves, and sequence and a short description of the spidroins used in this study (PDF)
