# Supplementary material for: Effects of Mini-Spidroin Repeat Region on the Mechanical Properties of Artificial Spider Silk Fibers
Source: ACS Omega. 2024 Oct 7;9(41):42423–32. doi: 10.1021/acsomega.4c06031 (PMC11483375; doi:10.1021/acsomega.4c06031)
Supplement: Supplementary file 1 — ao4c06031_si_001.pdf [file ao4c06031_si_001.pdf]

SUPPLEMENTARY INFORMATION

Effects of Mini-Spidroin Repeat Region on the Mechanical Properties of Artificial Spider Silk Fibers

Benjamin Schmuck[1,2], Gabriele Greco[2], Olga Shilkova[1], and Anna Rising[1,2*]*

[1] Department of Medicine Huddinge, Karolinska Institutet, Neo, 141 83 Huddinge, Sweden

[2] Department of Animal Bioscience, Swedish University of Agricultural Sciences, Uppsala, Sweden

KEYWORDS Protein-based fibers; Spidroin; High-performance fibers; Biomaterial; Optimization; Wet-spinning

* to whom correspondence should be addressed anna.rising@ki.se & benjamin.schmuck@ki.se

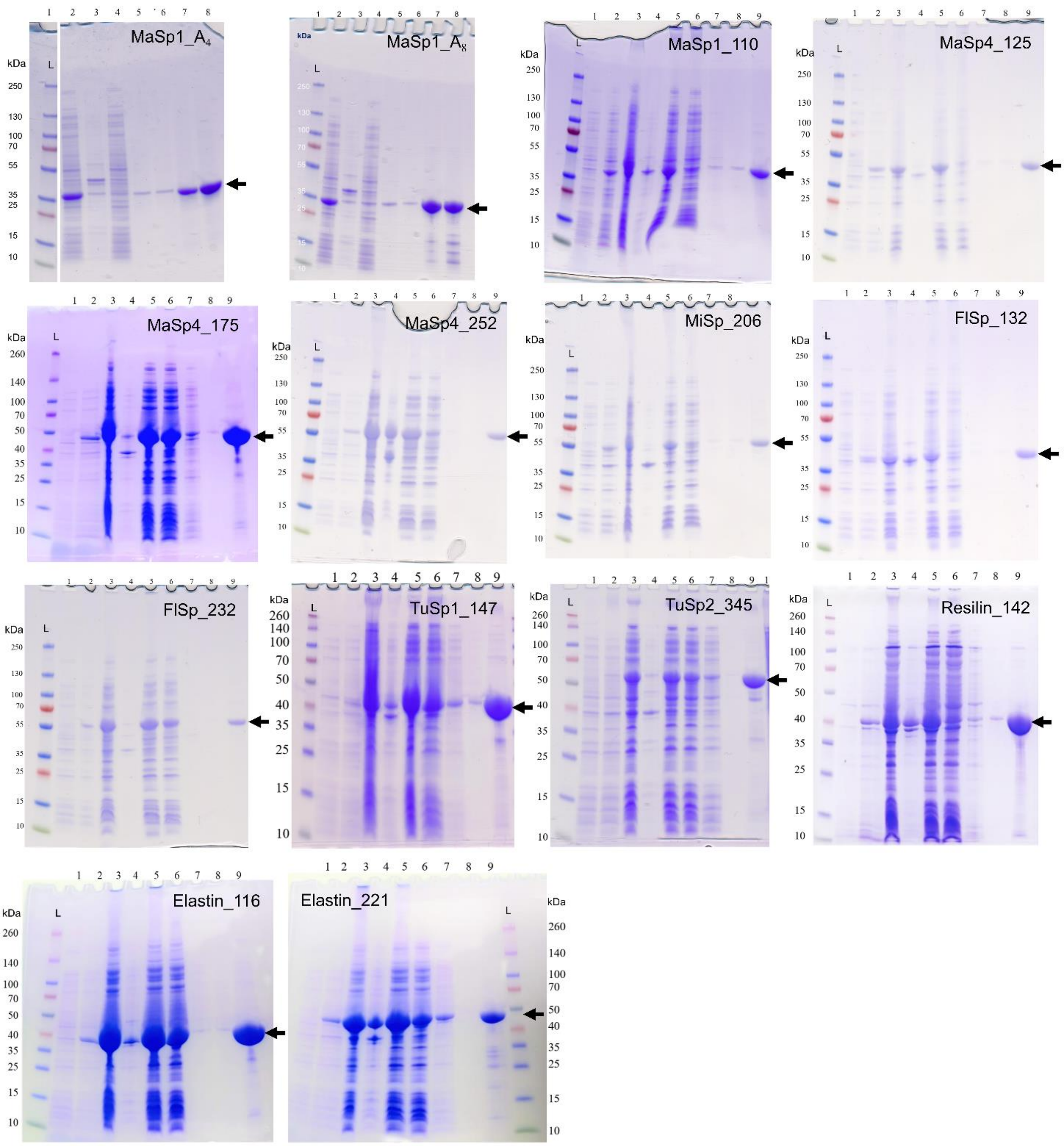

Figure S1. SDS-PAGE analysis of the expression and purification process of the different constructs described in this study. The arrows indicate the respective protein of interest. (L) indicates the protein size ladder and (kDa) the molecular masses of the bands. The lanes of the gel representing MaSp1_A_4 and MaSp1_A_8 show the following: (2) Supernatant after cell lysis and centrifugation containing all soluble proteins; (3) Insoluble debris after cell lysis; (4) IMAC Flow through; (5) IMAC Wash fraction 1; (6) IMAC Wash Fraction 2; (7) IMAC Elutate; (8) IMAC Eluate after dialysis. The lanes in all other gels represent the following samples: (1) Before induction; (2) after induction; (3) Total cell after resuspension of the cell pellet in lysis buffer; (4) Insoluble debris after cell lysis; (5) Supernantant after cell lysis and centrifugation containing all soluble proteins. (6) IMAC flow through; (7) IMAC Wash 1; (8) IMAC Wash 2; (9) Eluate after Dialysis.

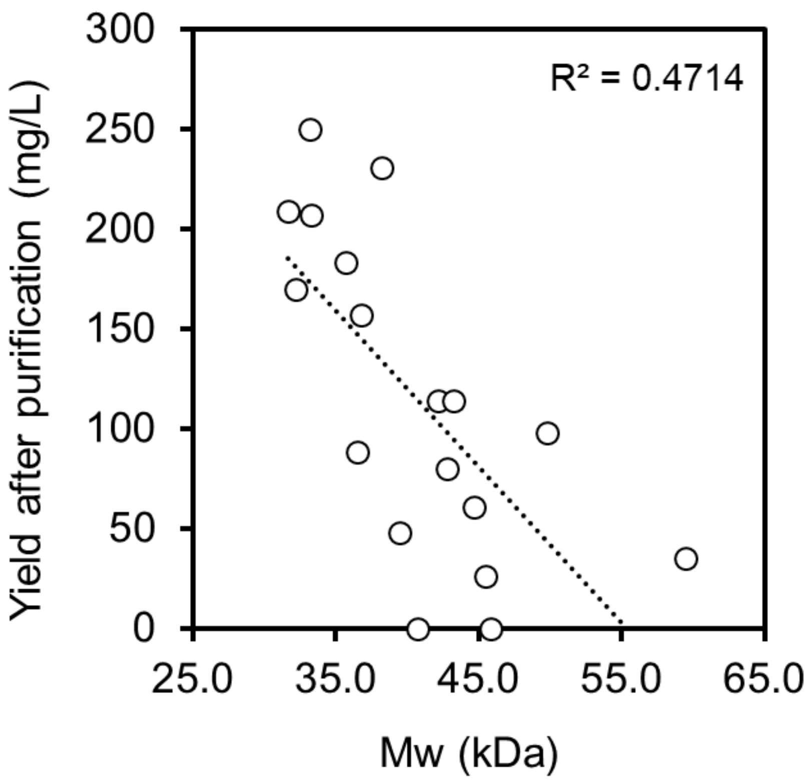

Figure S2. Shows the yield after purification as a function of the molecular weight. The dashed line shows the ordinary least square regression trend line ($p = 0.00165$).

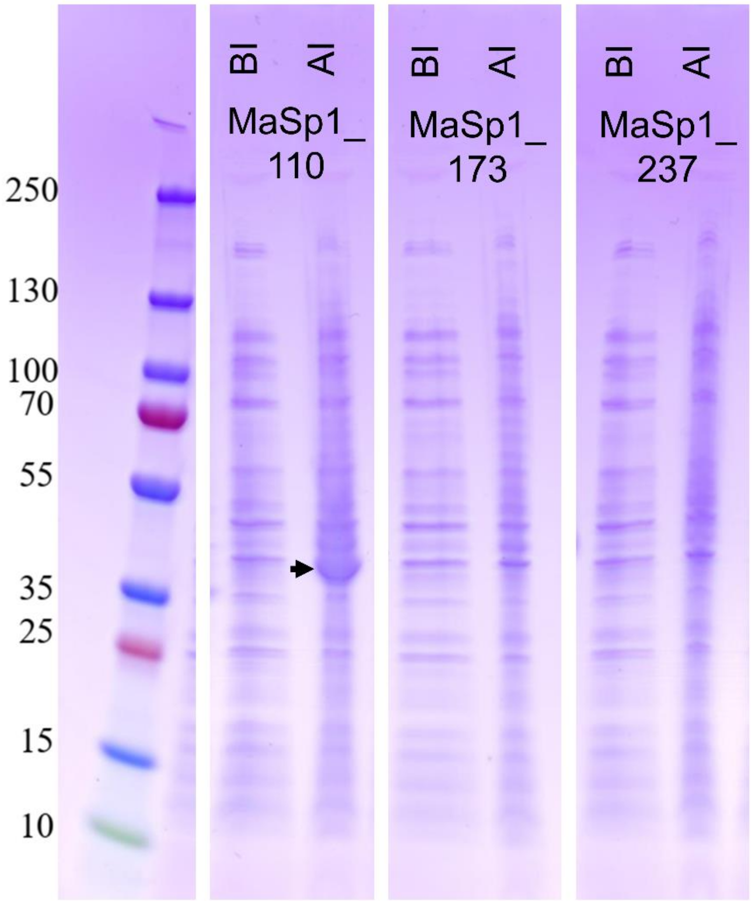

Figure S3. SDS PAGE comparing mini-spidroin constructs with Rep from MaSp1 before (BI) and after induction (AI). The samples loaded onto the gel were directly taken from the culture and are therefore representative for the total cell content. After induction, a new strong band is identified (indicated by an arrow) when using *E. coli* BL21 transformed with a vector containing a mini-spidroin with the MaSp1_110 insert. When the same strain was transformed with vectors containing mini-spidroins with MaSp1_173 and MaSp1_237 such a band was not seen. The left lane indicates the protein size ladder and number the molecular masses (kDa) of the bands.

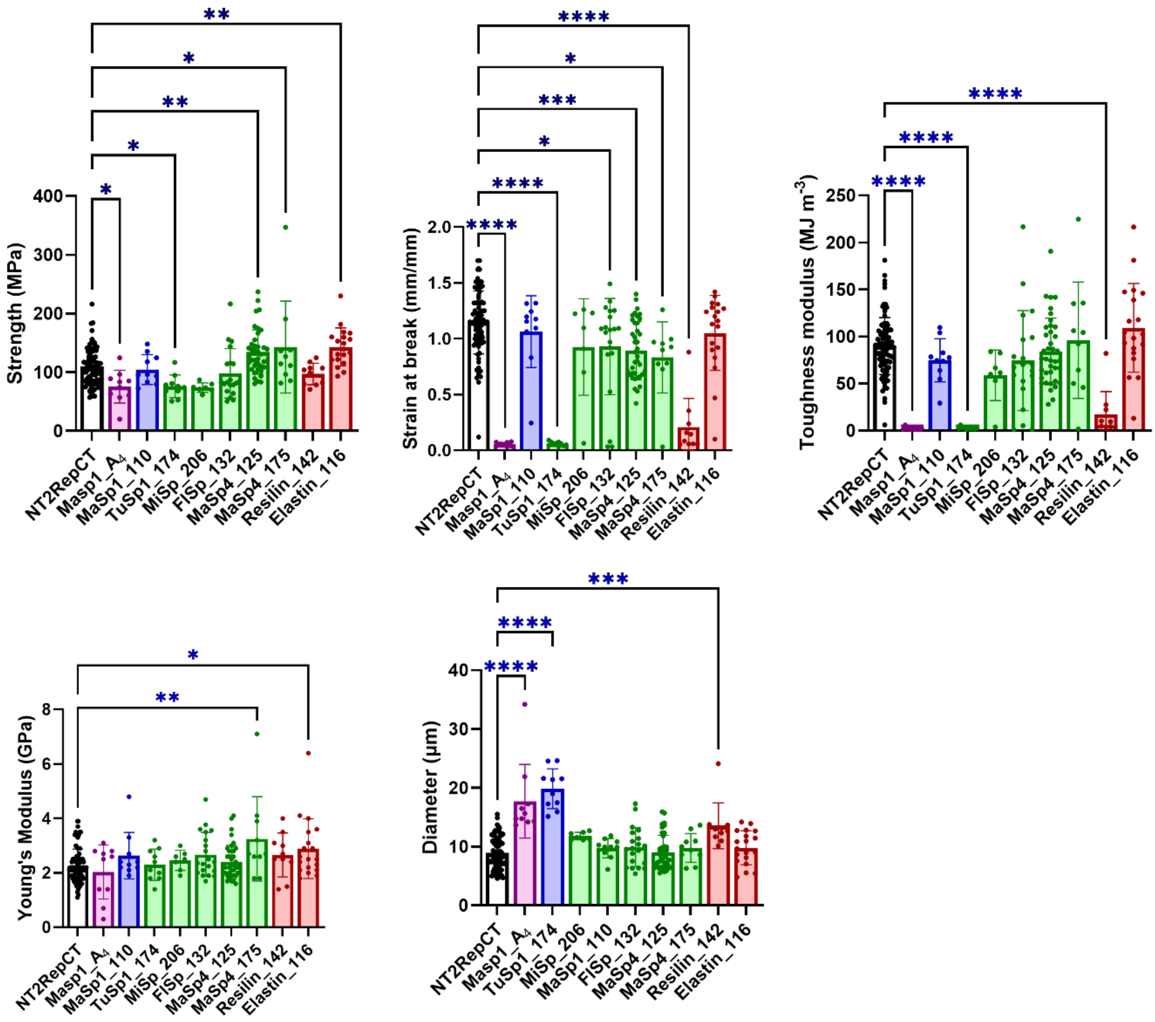

Figure S4. Graphical representation of the mechanical properties of the spinnable constructs as reported in **Table 2**. The box represents the average and the error bars show ± one standard deviation. A significant difference to NT2RepCT is indicated by an asterisk where * indicates $p \leq 0.05$, ** if $p \leq 0.01$, *** if $p \leq 0.001$, and **** if $p \leq 0.0001$. The boxes are colored according to the different spidroin engineering strategies as outlined in the discussion, where white was used for the control minispidroin, purple for the different length of the poly Ala, blue for the different lengths of MaSp1 Rep, green for variants where Rep originates from spidroins that are not MaSp1, and red for constructs that hosted a non-spidroin Rep. The graph was made using GraphPad Prism 10.

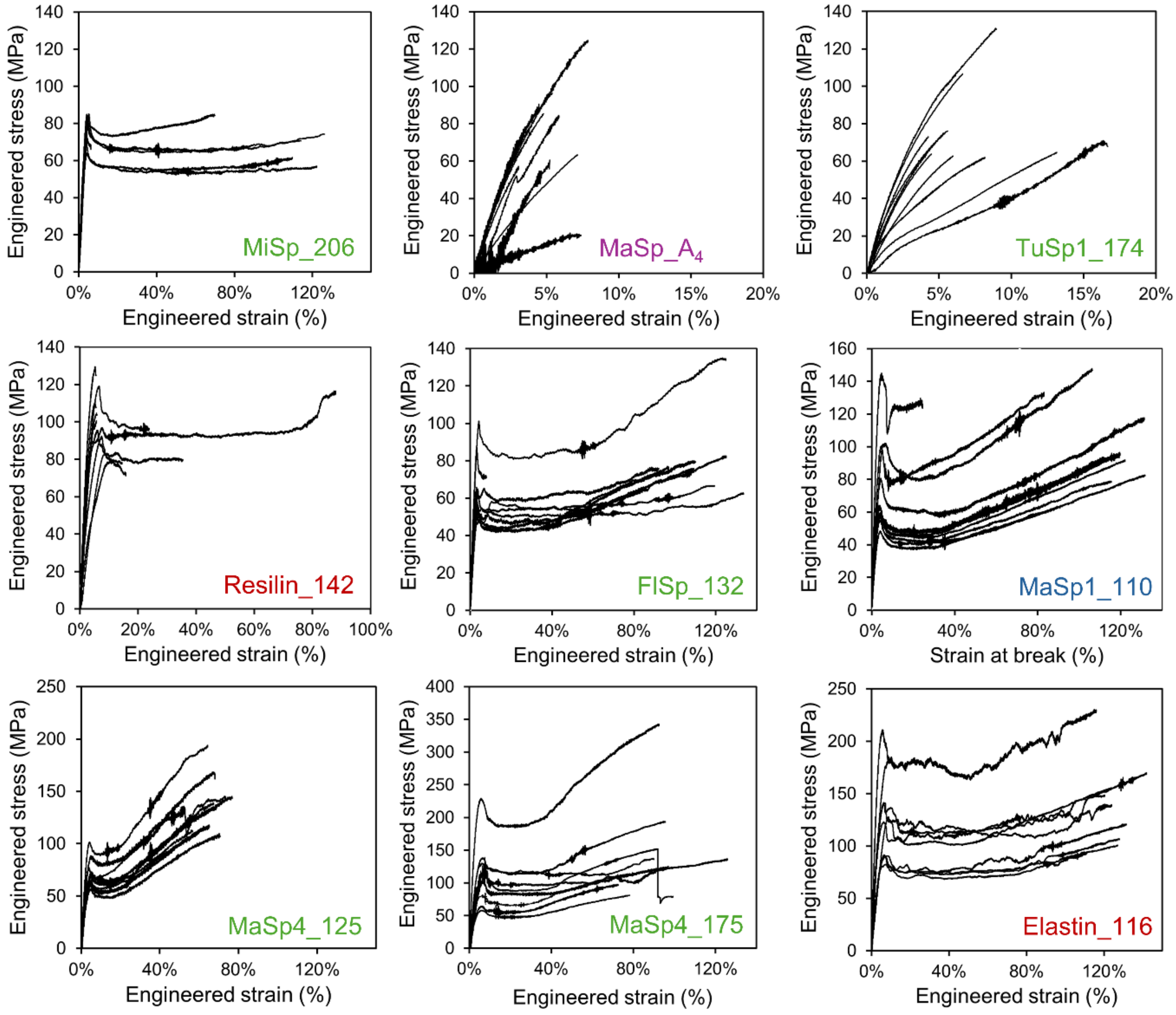

Figure S5. Representative stress-strain curves of the spinnable minispidroins described in this study, ordered in the same way as **Table 2**. The titles of each graph were colored according to the different spidroin engineering strategies in the same way as in **Figure S4**, where purple was used for the different length of the poly Ala, blue for the different lengths of MaSp1 Rep, green for variants where Rep originates from spidroins that are not MaSp1, and red for constructs that hosted a non-spidroin Rep.

Table S1. Collection of protein sequences and their corresponding molecular weight.

Domain	Sequence	Mw (Da)
His$_6$-NT[a,b]	MGHHHHHHMSHTTPWTNPGLAENFMNSFMQGLSSMPGFTASQLDDMSTIAQSMVQSIQSLAAQGRTSPNKLQALNMAFAS SMAEIAASEEGGGSLSTKTSSIASAMSNAFLQTTGVVNQPFINEITQLVSMFAQAGMNDVSAGNS	15265
CT[b]	SGSVTSGGYGYGTSAAAGAGVAAGSYAGAVNRLSSAEAASRVSSNIAAIASGGASALPSVISNIYSGVVASGVSSNEALI QALLELLSALVHVLSSASIGNVSSVGVDSTLNVVQDSVGQYVG	11592
2Rep (MaSp1_77)	GRGQGGYGQGSGGNAAAAAAAAAAAAAAAGQGGQGGYGRQSQGAGSAAAAAAAAAAAAAAGSGQGGYGGQGQGGYGQ	6342
MaSp1_A$_4$	GRGQGGYGQGSGGNAAAAGQGGQGGYGRQSQGAGSAAAAGSGQGGYGGQGQGGYGQ	4849
MaSp1_A$_8$	GRGQGGYGQGSGGNAAAAAAAAGQGGQGGYGRQSQGAGSAAAAAAAAGSGQGGYGGQGQGGYGQ	5418
(A$_3$I)$_3$-A$_{14}$	GRGQGGYGQGSGGNAAAIAAAIAAAIAAAGQGGQGGYGRQSQGAGSAAAAAAAAAAAAAAGSGQGGYGGQGQGGYGQ	6468
MaSp1_110	GGQGGQGQGGYGQGSGGSAAAAAAAAAAAAAAAGRGQGGYGQGSGGNAAAAAAAAAAAAAAAGQGGQGGYGRQSQGAGSA AAAAAAAAAAAAAGSGQGGYGGQGQGGYGQ	8886
MaSp1_173	GRGQGGYGQGAGGNAAAAAAAAAAAAAAGQGGQGGYGGLGQGGYGQGAGSSAAAAAAAAAAAAGGQGGQGQGGYGQGSGG SAAAAAAAAAAAAAAAGRGQGGYGQGSGGNAAAAAAAAAAAAAAAGQGGQGGYGRQSQGAGSAAAAAAAAAAAAAAGSGQ GGYGGQGQGGYGQ	13890
MaSp1_237	GRGQGGYGQGSGGNAAAAAAAAAAAASGQGSQGGQGGQGQGGYGQGAGSSAAAAAAAAAAAAASGRGQGGYGQGAGGNAA AAAAAAAAAAAAAAGQGGQGGYGGLGQGGYGQGAGSSAAAAAAAAAAAAGGQGGQGQGGYGQGSGGSAAAAAAAAAAAAA GRGQGGYGQGSGGNAAAAAAAAAAAAAAAGQGGQGGYGRQSQGAGSAAAAAAAAAAAAAAGSGQGGYGGQGQGGYGQ	19023
MaSp4_125	GPGPQGPSGPGPQGPYGPGPQGPGPQGPAPQGPSGPGPQRPQGPGPQRPYGPGGISVVSTTVGPGPQGPSAPGPQGPYGP GPQVPGPQGPGPQGPSGPGPQRPQGPGPQGPYGPGGVSVVSQTVS	11505
MaSp4_175	GPGPQGPSGPGPQGPYGPGPQGPGPQGPAPQGPSGPGPQRPQGPGPQRPYGPGGISVVSTTVSGPGPQGPSAPGPQGPYG PGPQVPGPQGPGPQGPSGPGPQRPQGPGPQGPYGPGGVSVVSQTVSGPGPQGPSGPGPQGPYGPGPQGPGPQGPGPQGPS GAGPQRPQGPGPQGP	15957
MaSp4_252	GPGPQGPLGPGAQVPYGPGPQVPGPQGPGPQGPSGPGPQRPQGPGPQGPYGPGGVSVVSQTVSGPGPQGPSGPGPQGPYG PGPQGPGPQGPAPQGPSGPGPQRPQGPGPQRPYGPGGISVVSTTVSGPGPQGPSAPGPQGPYGPGPQVPGPQGPGPQGPS GPGPQRPQGPGPQGPYGPGGVSVVSQTVSGPGPQGPSGPGPQGPYGPGPQGPGPQGPGPQGPSGAGPQRPQGPGPQGPYG PGGVSVVSATV	22925
MiSp_206	GAGAGGAGGYAQGYGAGAGAGAGAGTGAGGAGGYGQGYGAGSGAGAGGAGGYGAGAGAGAGAGGASGYGQGYGDGAGAGA GAAAAAGAAAGARGAGGYGGGAGDGAGAGAGAGAAGGYGQGYGAGEGAGAGAGGAGGYGAGAGAGGAGGYGQSYGDGA AAAAGSGAGAGGSGGYGAGAGAGAGAGAGSGAGAAGGYGGGAGAGV	15297
FlSp_132	GPGGYGPGGSGSGGYGPGGSGPGGSGPGGYGPGGTGPGGSESGGYGPGGSGPGGSGPGGSGPGGSGPGGYGPGGSGPSSF VPGGSGPGGSGPGGAGPGGAGPGGAGPGGAGPGGVGLGGAGRGGAGRGGAGS	10044
FlSp_232	GGSGPGGAGPGGAGPGGAGPGGAGPGGVGLGGAGRGGAGRGGAGSVGAGRGGAGRGGAGRGGAGRGGAGRGGAGGAGGAG GAGGPGGAGGSGGTTVIEDLDITIDGADGPITISEELTISGAGGSGPGGAGTGGVGPGGSGPGGVGPGGFGPGGVGPGGS GPGGVGPGGAGRPYGPGGSGPGGAGGAGGTGGAYGPGGAYGPGGSGGPGGAGGPGGEGPGGAGGPYGPGGAG	17884
TuSp1_174	SSSTTTTTSAARSQAASQSASSSYSSAFAQAASSSFATSSALSRAFSSVSSASAASSLAYSIGLSAARSLGIADAAGLAG ALARAVGALGQGATAASYGNALSTAAGQFFATAGLLNAGNASALASSFARAFSASAESQSFAQSQAFQQASAFQQAASRS ASQSAAEADSTSS	16422
TuSp2_345	SSSTTTTTIAARSQAASQSASSSYSSAFAQAASSSFATSSALSRAFSSVSSASAASSLAYSIGLSAARSLGIADAAGLAG ALARAVGALGQGATAASYGNALSTAAGQFFATAGLLNAGNASALASSFARAFSASAESQSFAQSQAFQQASAFQQAASRS ASQSAAEADSTSSSTTTTTSAARSQAASQSASSSYSSAFAQAASSSFATSSALSRAFSSVSSASAASSLAYSIGLSAARS LGIADAAGLAGALARAVGALGQGATAASYGNALSTAAGQFFATAGLLNAGNASALASSFARAFSASAESQSFAQSQAFQQ ASAFQQAASRSASQSAAEADSTSS	32678
Elastin_116	GGAGVPGVPGAIPGIGGIAGVGTPAAAAAAAAAAAKAAKYGAAAGLVPGGPGFGPGVVGVPGAGVPGVGVPGAGIPVVPGA GIPGAAVPGVVSPEAAAKAAAKAAKYGARPGVGVGG	9681
Elastin_221	GAAGKAGYPTGTGVGPQAAAAAAAKAAAKFGAGAAGVLPGVGGAGVPGVPGAIPGIGGIAGVGTPAAAAAAAAAAKAAKY GAAAGLVPGGPGFGPGVVGVPGAGVPGVGVPGAGIPVVPGAGIPGAAVPGVVSPEAAAKAAAKAAKYGARPGVGVGGIPT YGVGAGGFPGFGVGVGGIPGVAGVPSVGGVPGVGGVPGVGISPEAQAAAAAKAAKYGVGTP	18685
Resilin_142	GRPSSSYGAPGGGNGGRPSDTYGAPGGGNGGRPSDTYGAPGGGGNGNGGRPSSSYGAPGQGQGNGNGGRPSSSYGAPGGG NGGRPSDTYGAPGGGNGGRPSDTYGAPGGGNNGGRPSSSYGAPGGGNGGRPSDTYGAPGGGN	12661

a) The His$_6$-tag for purification is marked in green. b) Residues that originate from the cloning site are maked in red.

Table S2. Short description of nomenclature.

Name	Description
His_6-NT	N-terminal domain of MaSp1 from *Euprosthenops australis* with A His_6-tag. NT domain found in **NT**2RepCT
CT	C-terminal domain from *Araneus ventricosus* MiSp. CT domain found in NT2Rep**CT**
2Rep	short repetitive region from *E. australis* MaSp1 having 2 polyAla blocks. It has 77 residues in the Rep (MaSp1_77)
NT2RepCT	Previously characterized minispidroin made of His_6-NT and CT bracketing 2Rep – a control spidroin in this study
$MaSp1\_A_4$	Engineered short repetitive region from *E. australis* MaSp1 having 2 polyAla, but with only 4 Ala in both polyAla-blocks instead of 14 and 13.
$MaSp1\_A_8$	Engineered short repetitive region from *E. australis* MaSp1 having 2 polyAla, but having only 8 Ala in both polyAla-blocks instead of 14 and 13.
$(A_3I)_3$-A_{14}	Engineered variant of NT2RepCT, first descrined in Arndt 2022. Every 4th Ala in the first polyAla block was replaced with Ile. a control spidroin in this study
MaSp1_110	short repetitive region from *E. australis* MaSp1 having 3 polyAla. Longer variant of repetitive region found in NT2RepCT
MaSp1_173	short repetitive region from *E. australis* MaSp1 having 5 polyAla. Longer version of NT2RepCT
MaSp1_237	short repetitive region from *E. australis* MaSp1 having 7 polyAla. Longer version of NT2RepCT
MaSp4_125	Short Rep segment of Major ampullate spidroin 4 from *C. darwiini*
MaSp4_175	Rep segment of Major ampullate spidroin 4 from *C. darwiini,* longer than MaSp4 short
MaSp4_252	Rep segment of Major ampullate spidroin 4 from *C. darwiini,* longer than MaSp4 short
MiSp_206	Rep from Minor ampullate spidroin from Araneus ventricosus
FlSp_132	Rep segment from flagelliform spidroin from *Trichonephila clavipes*
FlSp_232	Rep segment from flagelliform spidroin from *Trichonephila clavipes,* longer than Flag
TuSp1_174	Rep from Tubulifrom spidroin 1 from *Trichonephila clavipes*
TuSp2_345	Rep from Tubulifrom spidroin 2 from *Trichonephila clavipes*
Elastin_116	Rep from Elastin from *Homo sapiens*
Elastin_221	Rep from Elastin from *Homo sapiens*
Resilin_142	Rep from Resilin from *Drosophila simulans*
